# Asthma under/misdiagnosis in primary care setting: an observational community-based study in Italy

**DOI:** 10.1186/s12948-015-0032-x

**Published:** 2015-11-16

**Authors:** Maria Sandra Magnoni, Marco Caminati, Gianenrico Senna, Fabio Arpinelli, Andrea Rizzi, Anna Rita Dama, Michele Schiappoli, Germano Bettoncelli, Gaetano Caramori

**Affiliations:** Medical and Scientific Department, GlaxoSmithKline, Verona, Italy; Allergy Unit, Verona University and General Hospital, Piazzale Stefani 1, 37126 Verona, Italy; Società Italiana di Medicina Generale, Florence, Italy; Dipartimento di Scienze Mediche, Sezione di Medicina Interna e Cardiorespiratoria, Centro Interdipartimentale per lo Studio delle Malattie Infiammatorie delle Vie Aeree e Patologie Fumo-Correlate (CEMICEF, formerly termed Centro), Università di Ferrara, Ferrara, Italy

**Keywords:** Asthma, ECRHS questionnaire, Under diagnosis, Misdiagnosis, Primary care

## Abstract

**Background:**

Published data suggest that asthma is significantly under/misdiagnosed. The present community-based study performed in Italy aims at investigating the level of asthma under/misdiagnosis among patients referring to the General Practitioner (GP) for respiratory symptoms and undergoing Inhaled corticosteroids.

**Methods:**

A sub-analysis of a previously published observational cross-sectional study has been provided. It included subjects registered in the GP databases with at least three prescriptions of inhaled or nebulised corticosteroids during the 12 months preceding the start of the study. All subjects, independently of the diagnosis, were invited to visit their GP’s office for a standardised interview and to fill the European Community Respiratory Health Survey (ECRHS) questionnaire.

**Results:**

The studies involved 540 GPs in most of the Italian regions and 2090 subjects (mean age 54.9 years, 54.1 % females) were enrolled. Among them 991 cases of physician-diagnosed asthma were observed while 1099 subjects received a diagnosis other than asthma (chronic obstructive pulmonary disease, chronic upper respiratory tract infections etc.). Among the lasts, the ECRHS questionnaire was suggestive for asthma diagnosis in 365 subjects (33.2 %).

**Conclusions:**

The data suggest that there is still a large under/misdiagnosis of asthma in the Italian primary care setting, despite the spread of GINA guidelines nearly 20 years before this study. A validated tool like the ECRHS questionnaire has detected a considerable proportion of potentially asthmatic patients who should be addressed to lung function assessment to confirm the diagnosis. Further educational efforts directed to the GPs are needed to improve their diagnosis of asthma (SAM104964).

## Background

A significant asthma under/misdiagnosis has been highlighted by some Italian studies. Ciprandi et al. [1] investigated the epidemiological features of asthma in a homogeneous population of 18-year-old male conscripts referred to La Spezia Military Navy Hospital for a call-up visit and found a not negligible under-diagnosis and inadequate treatment of asthma. In 7.4 % of conscripts asthma had been newly diagnosed during the study and about one quarter of the asthmatic subjects received no treatment at all. Bellia et al. [2] showed that asthma in the elderly is frequently confused with chronic obstructive pulmonary disease (COPD) and that in patients with mild functional impairment asthma may be under-diagnosed. A decreased perception of dyspnoea, or the intermittent onset of asthma symptoms, may account for under/misdiagnosis or delayed diagnosis of the disease [3]. Under/delayed-diagnosis and consequent under/delayed-treatment start might be important factors contributing to asthma morbidity, whereas early detection and treatment of asthma might improve the long-term prognosis of these patients.

The European Community Respiratory Health Survey (ECRHS) questionnaire has been proposed as a validated tool useful in identifying asthmatic patients [4–6]. The aim of the present community-based study was to investigate the level of asthma under/misdiagnosis in a primary care setting, by comparing physician diagnosis and the ECRHS questionnaire results. Patients undergoing inhaled corticosteroids (ICS) for a physician-diagnosed respiratory disease other than asthma were included as the study population. The results are reported as a sub-analysis of a previously published observational cross-sectional study [7].

## Methods

Full details of the study design and patient population have been reported elsewhere [7] and are summarised here.

### Study design

A multicentre, observational cross-sectional study involving of 540 Italian General Practitioners (GPs) has been conducted. The protocol was approved by the Ethic Committee of the Italian Society of General Medicine (SIMG; http://www.simg.it). Written informed consent was obtained by each patient before the inclusion into the study. Invitation to participate to the study was sent to all the GPs owning a computerised patient database according to the information stored in the archive of the European School of General Medicine (Scuola Europea di Medicina Generale, SEMG, Firenze, Italy).

Adult patients (≥18-years old) diagnosed with a respiratory disease and receiving at least 3 prescriptions of inhaled corticosteroids (ICS) during the previous 12 months (metered-dose inhaler -MDI-, dry powder inhaler -DPI- or nebulise) were enrolled. A concomitant prescription including long-acting beta2 agonists and/or theophylline and/or nedocromil or sodium cromoglycate, and/or antileukotrienes, and/or anticholinergic drugs was considered as an exclusion criterion, as usually these drugs are specifically and unequivocally prescribed for asthma or COPD.

This study was performed from September 2005 to January 2006. Every participating physician was requested to retrospectively select the last ten eligible consecutive patients since the study beginning date.

The selected patients were invited to perform a follow-up visit and to fill in an ECRHS respiratory symptom questionnaire. The first page contains validated questions on the presence of asthma and asthma-like symptoms, frequency of asthma attacks, age at onset and remission of asthma, doctor diagnosis of asthma, presence of chronic cough and phlegm, and smoking habits. The second page collects information on the last 12 months about: indirect costs (number of working days lost and number of impaired general activity days resulting from asthma); type and frequency of doctor visits and laboratory tests performed because of asthma; frequency of hospital admissions and emergency department (ED) visits resulting from asthma; treatment; type of prescription (when needed or for daily use) [4–6]. A subject with a questionnaire positive for respiratory symptoms (wheezing, nocturnal chest tightness, attack of breathlessness after activity at rest or at night; or 1 asthma attack) was considered a subject with current asthma.

## Results

Overall a response rate of 89 % was recorded corresponding to 2090 subjects (mean age 54.9 years, 54.1 % females). Among these subjects, according to the physician diagnosis 991 were affected by asthma and 1099 suffered from a respiratory disease other than asthma.

Table 1 shows demographic and clinical data of enrolled patients: comorbidities, such as cardiovascular diseases, are more frequently reported in patients with diagnosis other than asthma, whereas the prevalence of allergic disorders is higher in patients with asthma.

**Table 1:** Main characteristics of the patients with diagnosis with diagnosis other than asthma [N = 1099]

	N (%)	95 % IC
Mean age [years (SD)]	58.4 (18.3)	
Mean age at diagnosis of asthma (SD)	–	–
Female [n (%)]	590 (53.7)	50.6–56.6
Smoking habits		
Non smokers [n (%)]	614 (55.9)	52.8–58.8
Past smokers [n (%)]	199 (18.1)	15.8–20.5
Current smokers [n (%)]	277 (25.2)	22.6–27.8
Smoking history, mean years (SD)	(14.1) 10.8	–
Concomitant diseases		
Cardiovascular	445 (40.5)	37.5–43.4
Respiratory	119 (10.8)	9.0–12.8
Ear nose and throat (ENT)	171 (15.6)	13.4–17.8
Allergy	103 (9.4)	7.7–11.2
Spirometry	181 (16.5)	14.3–18.7

% accounting also for missing data

Missing data: smoking habits = 20

In patients diagnosed with a respiratory disease other than asthma, COPD was the most frequently reported (21.7 %), followed by not specified upper respiratory tract infections (12.2 %), chronic or acute bronchitis (11.5 %). Overall, upper respiratory symptoms and/or signs were present in around 40 % of these patients, classified as allergic or vasomotor rhinitis, chronic otitis/sinusitis, not specified otitis/sinusitis, not specified rhinitis, not specified acute upper respiratory tract infections and not specified upper respiratory tract infections. In 4.9 % other different respiratory diseases were reported.

**Fig. 1:**
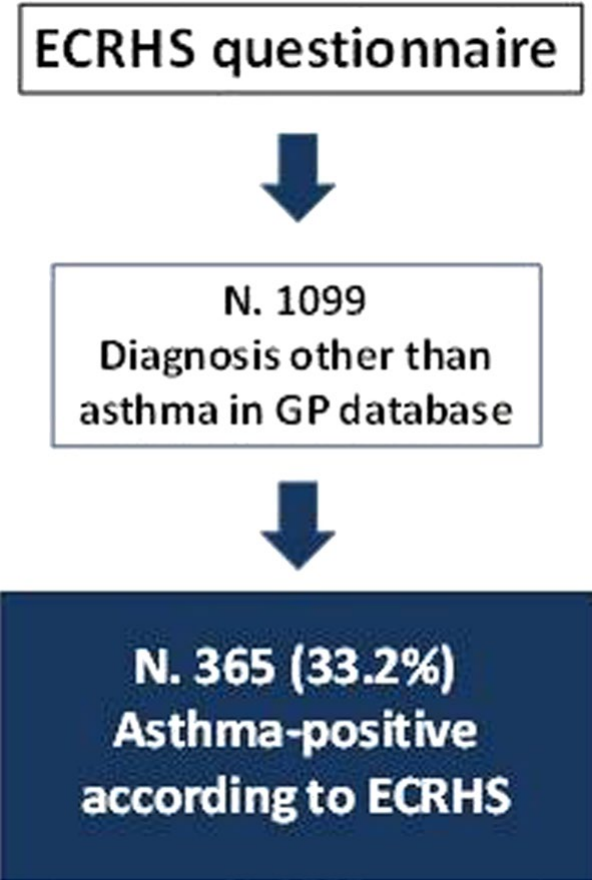
Results from the ECRHS questionnaire administered to patient with respiratory symptoms and diagnosis other than asthma in the GP database

Among patients diagnosed with a respiratory disease different from asthma (1099), the ECRHS asthma questionnaire suggested asthma diagnosis in 365 (33.2 %) of them (Fig. 1). The characteristics of this subgroup are reported in Table 2. Most diagnoses (about 60 %) were related to chronic obstructive lung diseases, bronchitis (chronic or acute bronchitis), whereas around 20 % were related to high respiratory airways (acute or chronic upper respiratory tract infections, allergic or vasomotor rhinitis, otitis/sinusitis). Of note, only 16.5 % of patients had undergone lung function assessment in the last 12 months. In particular, less than 30 % of asthmatic patients according to the ECRHS questionnaire and 38.6 % of physician-diagnosed asthmatic patients underwent spirometry (Table 1).

**Table 2:** Diagnosis reported in the GP data base of the 365 subjects identified as asthmatics by ECRHS questionnaire

Diagnosis	Patients (n)	%
COPD	128	35.1
Chronic bronchitis or acute bronchitis	53	14.5
Respiratory symptoms or signs	47	12.9
Upper respiratory tract infections (n.s.)	21	5.8
Acute bronchitis	20	5.5
Bronchitis n.s.	15	4.1
Allergic or vasomotor rhinitis	11	3.0
Chronic upper respiratory tract infections	10	2.7
Chronic otitis/sinusitis	10	2.7
Acute upper respiratory tract infections (n.s)	10	2.7
Otitis/sinusitis (n.s.)	8	2.2
Other respiratory diseases	32	8.8
Total	365	100.0

*n*.*s*. not specified

## Discussion

Our study highlights that according to the ECRHS results, asthma should be highly suspected in 33.2 % in patients diagnosed with a respiratory disease different from asthma by their GP and undergoing ICS treatment. Furthermore only 16.5 % of the overall study population had undergone lung function assessment in the last 12 months, despite suffering from a physician-diagnosed respiratory disease.

The results of our study show a poor accordance between physician-reported and ECRHS questionnaire-related asthma diagnosis. Assuming the good sensibility and specificity of the ECRHS questionnaire, the results confirm that Italian GPs do not optimally recognise respiratory symptoms as asthma manifestations. This finding is in agreement with previous studies, showing that 7.4 % of enrolled subjects had been newly diagnosed with asthma during the study and about one quarter of the asthmatic subjects received no treatment at all [1].

It is not surprising that in our cohort of patients, selected on the basis of ICS use, many of them had a diagnosis different from asthma. In Italy, patients with COPD, acute and chronic bronchitis, acute upper respiratory tract infections, rhinitis or not well defined respiratory symptoms are extensively treated with ICS, as also reported in other studies, including a large-scale paediatric survey [8].

Among the patients identified as asthmatics according to ECRHS questionnaire, 35 % had a diagnosis of COPD in GP database, although around 56 % of them had never smoked. Distinguishing asthma from COPD is often problematic, particularly in smokers and older adults, and in a significant proportion of patients COPD and asthma features may coexist [9]: spirometry, besides clinical history, could help to address the question of differential diagnosis, and it should always be performed in patients with respiratory symptoms. Nevertheless in our study less than 30 % of asthmatic patients according to the ECRHS questionnaire and 38.6 % of physician-diagnosed asthmatic patients underwent lung function assessment during the previous 12 months. Limited prescription of lung function tests in general practice (physicians may not be fully familiar with the interpretation of results) and poor accessibility to spirometers, which are mostly available in the hospital setting due to lack of time to perform office spirometry (in most cases GPs in Italy do not have technical or nursing support), may account for under-utilization of spirometry in primary care [10, 11].

Around 20 % of the patients identified as asthmatics according to ECRHS questionnaire had a diagnosis of upper airway disease in the GP database (allergic or vasomotor rhinitis or otitis/sinusitis). It is well known that allergic rhinitis and sinusitis are often associated with asthma and constitute the main risk factor for its development. Another Italian study showed that subjects with allergic rhinitis show an eightfold risk of having asthma compared to subjects without allergic rhinitis [12]. Furthermore, in a large cohort study on subjects with allergic rhinitis without diagnosis of asthma, bronchial hyper-responsiveness and also bronchial obstruction were detected in a high percentage of patients, both during and outside the pollen season [13], underlining the importance of lung function assessment in patients with chronic upper airways symptoms. Nevertheless the lack of asthma identification in these patients suggests that asthma is still regarded mainly as an intermittent disease, or misrecognized as a clinical manifestation of viral infections.

Our study has some potential limitation. Firstly, only patients on ICS treatment were included in the survey, whereas those treated with other respiratory drugs were excluded. Although inhaled corticosteroids are the gold standard of asthma therapy, in general practice there is a wide range of treatments for patients with respiratory symptoms. Thus, the rate of mis/underdiagnosis of asthma observed in this study presumably affects milder patients. As regards patients treated with various anti-asthmatic agents, such as combinations of ICS and bronchodilators, recent evidence suggests a considerable amount of overdiagnosis of asthma [14]. Secondly, untreated patients were excluded from the study population, thus patients with milder disease have been potentially lost.

Our data suggest that there is still a considerable under/misdiagnosis of asthma in the Italian primary care settings, and that the use of a validated questionnaire could be of helpful in identifying patients to address to lung function assessment.

## Conclusions

Asthma under/misdiagnosis and consequent inappropriate pharmacological treatment still affect asthma management. Furthermore they represent important factors contributing to asthma morbidity and mortality, whereas early detection and management might improve the long-term prognosis of affected patients. Educational efforts should be directed to improve the capability of primary care professionals, particularly GPs, to recognise asthma symptoms and to address patients to the correct diagnostic work-up and proper treatment. The use of a validated questionnaire could be of help for patients’ identification.

## Abbreviations

GP: General Practitioner
ECRHS: European Community Respiratory Health Survey
COPD: chronic obstructive pulmonary disease
ICS: inhaled corticosteroids
MDI: metered-dose inhaler
DPI: dry powder inhaler
ED: emergency department

## References

[1] Ciprandi G, Vizzaccaro A, Cirillo I, Tosco M, Passalacqua G, Canonica GW (2001). Underdiagnosis and undertreatment of asthma: a 9-year study of Italian conscripts. Int Arch Allergy Immunol.

[2] Bellia V, Battaglia S, Catalano F, Scichilone N, Incalzi RA, Imperiale C (2003). Aging and disability affect misdiagnosis of COPD in elderly asthmatics. The SARA Study. Chest.

[3] van Weel C (2002). Underdiagnosis of asthma and COPD: is the general practitioner to blame?. Monaldi Arch Chest Dis.

[4] de Marco R, Cerveri I, Bugiani M, Ferrari M, Verlato G (1998). An undetected burden of asthma in Italy: the relationship between clinical and epidemiological diagnosis of asthma. Eur Respir J.

[5] de Marco R, Zanolin ME, Accordini S, Signorelli D, Marinoni A, Bugiani M (1999). A new questionnaire for the repeat of the first stage of the European Community Respiratory Health Survey: a pilot study. Eur Respir J.

[6] de Marco R, Bugiani M, Cazzoletti L, Carosso A, Accordini S, Buriani O, et al, for ISAYA study Group. The control of asthma in Italy. A multicentre descriptive study on young adults with doctor diagnosed current asthma. Allergy. 2003; 58:221–228. 10.1034/j.1398-9995.2003.00059.x 12653796

[7] Caminati M, Bettoncelli G, Magnoni MS, Rizzi A, Testi R, Passalacqua G (2014). The level of control of mild asthma in general practice: an observational community-based study. J Asthma.

[8] Clavenna A, Rossi E, Berti A, Pedrazzi G, De Rosa M (2003). Bonati M; ARNO Working Group. Inappropriate use of antiasthmatic drugs in the Italian paediatric population. Eur J Clin Pharmacol.

[9] The Global Strategy for Asthma Management and Prevention Report 2015. http://www.ginasthma.org/local/uploads/files/GINA_Report_2015_May19.pdf. Accessed 30 May 2015.

[10] Lusuardi M, De Benedetto F, Paggiaro P, Sanguinetti CM, Brazzola G, Ferri P (2006). A randomised controlled trial on office spirometry in asthma and COPD in standard general practice: data from spirometry in asthma and COPD. A comparative evaluation Italian Study. Chest.

[11] Caramori G, Bettoncelli G, Tosatto R, Arpinelli F, Visonà G, Invernizzi G (2005). Underuse of spirometry by general pratictioners for the diagnosis of COPD in Italy. Monaldi Arch Chest Dis.

[12] Bugiani M, Carosso A, Migliore E, Piccioni P, Corsico A, Olivieri M (2005). Allergic rhinitis and asthma comorbidity in a survey of young adults in Italy. Allergy.

[13] Cirillo I, Vizzaccaro I, Tosca MA, Negrini S, Negrini AC, Marseglia GL (2005). Bronchial hyperreactivity and spirometric impairment in patients with allergic rhinitis. Monaldi Arch Chest Dis.

[14] Heffler E, Pizzimenti S, Guida G et al. Prevalence of over-/misdiagnosis of asthma in patients referred to an allergy clinic. J Asthma. 2015;1–4. 10.3109/02770903.2015.1026442 26291138

